# Low frequency of disease flare in patients with rheumatic musculoskeletal diseases who received SARS-CoV-2 mRNA vaccine

**DOI:** 10.1186/s13075-021-02674-w

**Published:** 2022-01-11

**Authors:** Francesca Romana Spinelli, Ennio Giulio Favalli, Cristina Garufi, Martina Cornalba, Serena Colafrancesco, Fabrizio Conti, Roberto Caporali

**Affiliations:** 1 Department of Clinical Internal, Anaesthesiologic and Cardiovascular Sciences – Rheumatology Unit, Sapienza University of Rome, Rome, Italy; 2 Division of Clinical Rheumatology, ASST Gaetano Pini – CTO Institute, Milano, Italy; 3 Department of Clinical Sciences & Community Health, Research Center for Adult and Pediatric Rheumatic Diseases, Università degli Studi di Milano, Milano, Italy

**Keywords:** Vaccine, Rheumatic musculoskeletal disease, SARS-CoV-2, COVID-19

## Abstract

**Background:**

Little is known about the safety of SARS-CoV-2 vaccination in patients with rheumatic musculoskeletal disease (RMD). We evaluated the occurrence of adverse events following immunization (AEFI) in RMD patients and heathy subjects who received anti-SARS-CoV-2 mRNA vaccine.

**Methods:**

We performed a telephone interview collecting any adverse event (AE) following immunization (AEFI) that occurred in RMD patients and healthy controls after the two doses of mRNA vaccine including common local reactogenicity and systemic events (for example, fever, fatigue/malaise, joint and muscle pain). We also investigated the onset of new signs or symptoms of the RMD after the vaccination.

**Results:**

We evaluated 126 patients with RMDs [105 females and 19 males, median age 51(IQR 17)] and 85 controls [62 females and 23 males, (median age 49 (20)]. Seventy patients (55.6%) were taking immunosuppressants, conventional synthetic (*n*=31, 43.3%) and/or biological [TNF inhibitors (*n*=49, 68.6%)], and 30 (23.8%) were taking hydroxychloroquine; treatment remained unchanged in 77% of patients. Eleven out of 126 patients and none of the 85 controls previously contracted COVID-19. The median follow-up from the completion of vaccination was 15 (3) weeks both in patients and controls. We reviewed 5 suspected cases confirming mild articular flares in 3 women (2.8) with inflammatory arthritis (2 psoriatic arthritis and 1 rheumatoid arthritis) while no disease reactivation was recorded in patients with connective tissue diseases; the incidence rate of RMD reactivation was 0.007 person/month. Multivariable logistic regression analysis showed similar frequencies of local and systemic AEFI in patients and controls with no effect of therapies or previous COVID-19. Local reaction—pain in the injection site—was the most frequently reported AEFI both in RMD and controls (71% and 75% of all the AEFI, respectively) after the first dose. Overall, up to 66% of patients experienced at least one AEFI at the second dose and up to 62% in the control group. Most of AEFI occurred within 2 days of vaccine administration. Two RMD patients developed pauci-symptomatic COVID-19 after the first dose of vaccine.

**Conclusion:**

The low incidence rate of disease reactivation and the similar AEFI occurrence compared to controls should reassure on mRNA vaccine safety in RMD patients.

**Supplementary Information:**

The online version contains supplementary material available at 10.1186/s13075-021-02674-w.

## Background

In January 2021, the vaccine campaign against COVID-19 has begun, starting from the healthcare professionals. Up to October 2021, about 70% of the Italian adult population (> 12 years) has completed the vaccination. Meanwhile, people diagnosed with rheumatic musculoskeletal diseases (RMD) began asking about the safety of anti-SARS-CoV-2 vaccine in relation to their condition. However, at the time of the beginning of the vaccination campaign, data from only a few dozen patients included in randomized clinical trials (RCTs) were available and seemed reassuring. Still today, people raise concerns about vaccination, related to both efficacy and safety—in terms of adverse events and, most of all, about the possible reactivation of the underlying disease. A recent report evaluating the adverse reactions occurred after the first dose of mRNA vaccine, either Pfizer BioNTech or Moderna, showed a frequency of local and systemic reactions consistent to that reported in the clinical trials [1–3]. Up to now, few data has been reported on the safety profile of the two doses of mRNA SARS-CoV-2 vaccination in patients with RMD. Therefore, in this study, we aimed at evaluating the short-term safety of anti-SARS-CoV-2 vaccine in patients with RMD in terms of both adverse events and disease flare-up.

### Patients and methods

This observational study was performed at the Rheumatology Units of Sapienza University of Rome and Gaetano Pini Institute, Milan. We recruited health care professionals diagnosed with RMD (hereinafter referred as “patients”) regularly followed up at our Rheumatology Units, who received the SARS-COV-2 mRNA vaccine first at the beginning of the vaccination campaign. Eligible subjects were identified by searching outpatient clinics databases; RMD patients who contacted their rheumatologists for advice before getting vaccinated were also enrolled. As healthy controls (HC), we included healthcare professionals who were not diagnosed with any RMD and were not taking glucocorticoids or immunosuppressant drugs for any health conditions. Healthy controls were identified from the registry of healthcare workers who underwent the vaccination at the two centers involved in the study. Only one patient, and no control subject, declined to participate to the telephone interview. All participants answered the entire interview, so there were no missing data. Subjects who gave their consent to participate to the study were recalled by phone by the authors (FRS, EGF, CG, and MC), 1 week after the first and 2 weeks after the second vaccine dose; subjects were instructed to report the emergence of any possible signs and symptoms afterwards up to May 2021. Supplementary Figure 1 reports the flowchart of phone interviews. We collected any adverse event (AE) following immunization (AEFI) that occurred after the two doses of mRNA vaccine including common local reactogenicity (in particular, pain, redness, and swelling in the injection site) and systemic events (for example, fever, fatigue/malaise, joint and muscle pain). We also investigated the onset of new signs or symptoms of their RMD that occurred after the vaccination and any action taken to control the eventual disease flare. The causality relationship between vaccination and disease flare was discussed by 4 rheumatologists of the two teams according to the World Health Organization algorithm for AEFI [4]. In suspected cases, the time-lapse between vaccination and the onset of symptoms clearly attributable to the underlying RMD, the type of clinical manifestation, and symptoms duration were considered. Data were collected in a dedicated electronic case report form and included sex, age, diagnosis, disease duration, and ongoing therapy at the time of vaccination. Verbal or written informed consent to use their anonymized data was obtained from patients and controls. The protocol was approved by the Local Ethical Committee (prot. 0501/2021).

Data were expressed as median and interquartile range (IQR); frequencies and proportions were reported for continuous or categorical variables, respectively. Mann-Whitney and chi-square tests were used to compare the statistical significance of differences in the distribution of continuous or categorical variables, respectively, between RMD patients and HC. To account for baseline clinical differences among RMD patients and controls, multivariable logistic regression analysis was used to assess the impact of the presence of a RMD on the occurrence of AEFI. Multivariable logistic regression was also used to assess the impact of specific features of RMD patients (i.e., ongoing therapy, previous SARS-CoV-2 infection, and suspension of therapy before vaccination) on AEFI occurrence. Finally, multivariable logistic regression was applied to evaluate the impact of previous SAR-CoV-2 infection, treatment for RMD, and RMD itself on the occurrence of systemic versus local AEFI. For all models, we selected covariates representing the most relevant features possibly acting as confounders, i.e., age and sex. The possibility of an interaction between such predictors was also tested by the introduction of interaction terms at logistic regression analysis.

All statistical tests were performed using the RStudio graphical interface v.0.98 for R software environment v.3.0.2. All tests were two-sided with a significance level set at *p*<0.05.

## Results

Between 30 December 2020 and 22 February 2021, we evaluated 126 patients with RMDs [median age 51 (17), 105 females and 19 males, percentage of females 84%] and 85 healthy controls [median age 49 (20), 62 females and 23 males, percentage of females 72.9%; *p*=ns compared to RMD patients]. The median follow-up from the completion of vaccination was 15 (3) weeks.

Table 1 summarizes the demographic and clinical data of enrolled subjects. Most of the patients (*n*=105, 83.3%) were taking at least one drug for their disease. Seventy patients (55.6%) were taking immunosuppressants, synthetic [methotrexate (*n*=21, 30%), other *n*=10 (14.3%)] and/or biological [TNF inhibitors (*n*=34, 48.6%), other (*n*=15, 21.4%)]; 30 patients (23.8%) were taking hydroxychloroquine. Three patients were treated with JAK-inhibitors and one patient with apremilast. At the time of vaccination, treatment remained unchanged in 98 out of 126 patients (77.8%). Eleven patients (8.7%), and none of the controls, had been previously  diagnosed with COVID-19.

**Table 1:** Demographic and clinical data of subjects participating to the study

	Patients (*n*=126)	Controls (*n*=85)	*p*
Female:male (% female)	105:19 (84)	62:23 M (72.9)	0.06
Age [median (IQR)], years	51 (17)	49 (20)	0.11
Rheumatoid arthritis, *n* (%)	31 (24.6)		
Systemic lupus erythematosus, *n* (%)	31 (24.6)		
Psoriatic arthritis, *n* (%)	26 (20.6)		
Undifferentiated connective tissue disease, *n* (%)	11 (8.7)		
Ankylosing spondylitis, *n* (%)	9 (7.1)		
Other^a^, *n* (%)	18 (14.3)		
Glucocorticoids, *n* (%)	28 (22.2)		
Hydroxychloroquine, *n* (%)	30 (23.8)		
Immunosuppressive drugs	70 (55.6)		
Methotrexate, *n* (%)	21 (16.6)		
Other csDMARDs, *n* (%)	10 (7.9)		
TNF inhibitors, *n* (%)	34 (27)		
Other b/tsDMARDs, *n* (%)	15 (11.9)		
Stopped ongoing therapy for vaccination, *n* (%)	28 (22.2)		

^a^Other diagnoses included Sjogren syndrome (*n*=4), scleroderma (*n*=3), mixed connective tissue diseases (*n*=2), systemic vasculitis (*n*=4), and anti-phospholipid syndrome (*n*=5)

*bDMARDs* biological disease modifying anti-rheumatic drugs, *csDMARDs* conventional synthetic disease modifying anti-rheumatic drugs, *TNFi* tumor necrosis factor inhibitor

We reviewed 5 suspected cases confirming a disease flare in 3 (2.8%) women with inflammatory arthritis (2 psoriatic arthritis and 1 rheumatoid arthritis). None of the patients with connective tissue diseases or vasculitis reported disease flare. All disease reactivations consisted in mild articular flares lasting 7 days on average and requiring just symptomatic treatment. None of the 3 patients stopped treatment before vaccination. All disease reactivations were mild articular flare (case #1 mono-arthritis of the left proximal interphalangeal joint, case #2 inflammatory polyarthralgia, case #3 inflammatory back and neck pain) lasting 7 days on average and requiring just symptomatic treatment with glucocorticoids (case #2) or non-steroidal anti-inflammatory drugs (#case 3). None of the three patients stopped ongoing treatment before vaccination. Clinical details of patients experiencing disease reactivation are reported in Table 2.

**Table 2:** Clinical details of patients experiencing disease reactivation

	Patient #1	Patient #2	Patient #3
Sex	F	F	F
Age	47	60	58
RMD diagnosis	PsA	RA	PsA
Ongoing treatment	None	MTX	TNFi
Type of flare	III PIP arthritis	Inflammatory polyarthralgia	Inflammatory back and neck pain
Days from II dose of vaccine	18	10	3
Action taken	None	Glucocorticoids (4 days)	NSAID (once)
Outcome	Resolved in 10 days	Resolved in 10 days	Resolved in 3 days

*PsA* psoriatic arthritis, *RA* rheumatoid arthritis, *MTX* methotrexate, *TNFi* tumor necrosis factor inhibitor, *PIP* proximal interphalagneal, *NSAIDs* non-steroidal anti-inflammatory drug

Among the 126 patients enrolled in the study, in up to 5 months of follow-up, we detected an incidence rate of RMD reactivation of 0.007 person/month.

**Fig. 1:**
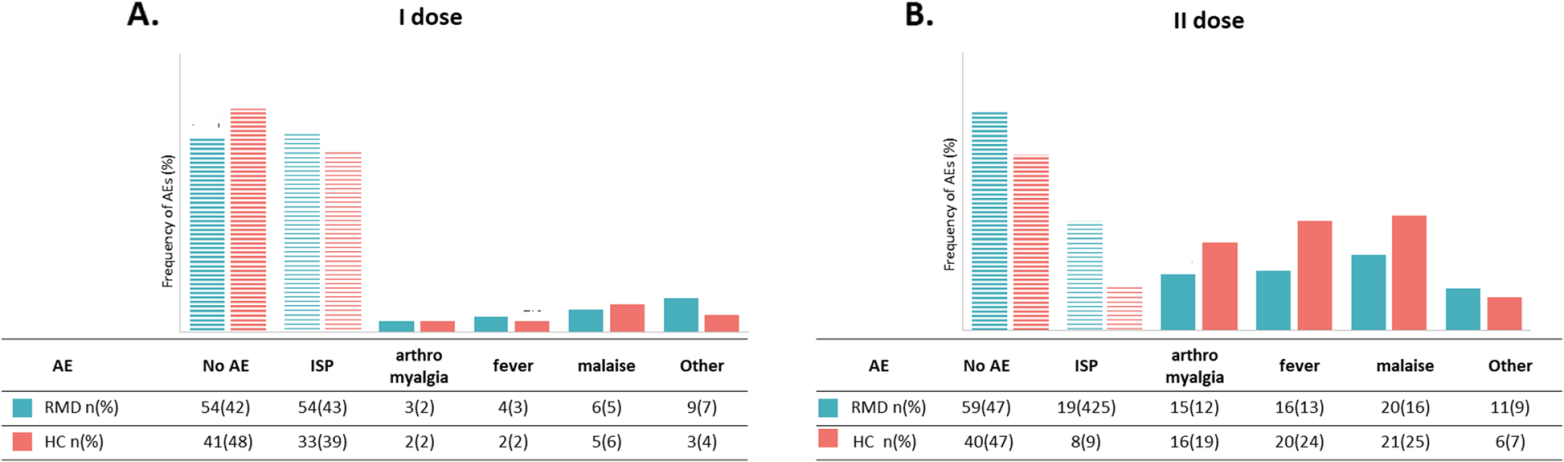
Local reactions and systemic events in patients with RMD and healthy controls after the first and second dose of vaccine. Other adverse events included headache, nausea, diarrhea, hypertension, and lymphadenopathy. Frequency of different adverse events after first (**A**) and second (**B**) dose of vaccine. AE, adverse events; HC, healthy controls; RMD, rheumatic musculoskeletal disease

The type and frequency of different reactions are reported in Fig. 1A, B.

At univariate analysis, local reaction—pain in the injection site—was the most frequently reported AEFI after the first dose both in RMD and controls (71% and 75% of all the AEFI, respectively) without any difference at multivariable regression analysis. After the second dose, the percentage of systemic reactions increased in both groups, representing 67% of all the AEFI in RMD patients and 80% in HC (*p*=ns).

**Fig. 2:**
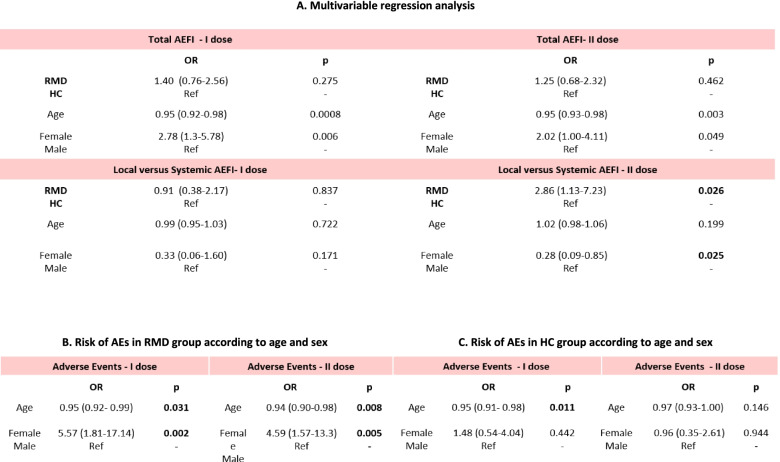
Risk of any adverse events following immunization in RMD patients and controls according to age and sex. AEFI, adverse events following immunization; HC, healthy controls; RMD, rheumatic musculoskeletal disease

At univariate analysis, a similar percentage of patients and controls experienced at least one AEFI after the first dose of vaccine [76/126 (60.3%) vs 44/85 (52%), respectively; *p*=ns)] and after the second dose [80/126 (65%) vs 52/85 (62%), respectively; *p*=ns)]. Multivariable logistic regression analysis, using predictors “presence of RMD,” “age,” and “sex,” confirmed no difference in AEFI occurrence between RMD and HC both following the first and following the second dose (Fig. 2A).

After the first dose, local reaction was the most frequently reported AEFI both in RMD and controls [54/126 (43%) and 33/85 (39%), *p*=ns]. Multivariable logistic regression analysis, using predictors “presence of RMD,” “age,” and “sex,” showed no difference in local AEFI between RMD vs HC (Fig. 1C). Similarly, the percentage of patients and controls reporting the various systemic AEFIs after the first dose did not differ (supplementary Figure 2).

After the second dose, univariate analysis showed a numerically higher prevalence of local reactions in RMD patients compared to HC [19/126 (23%) vs 8/85 (9%), *p*=ns] and a numerically lower frequency of systemic AEFI [54/126 RMD (43%) vs 42/85 HC (49%), *p*=0.348].

Multivariable logistic regression analysis, using “presence of RMD,” “age,” and “sex” as predictors, showed a higher risk of local than systemic AEFI in RMD patients (Fig. 2A). Moreover, the frequency of fever after the second dose was significantly lower in RMD patients compared to HC (OR=0.38 *p*=0.030) (Supplementary Figure 2).

“Most of AEFI occurred within the first two days after the administration of the first and second dose both in RMD patients (85.5% and 82%, respectively) and HC (100% after both doses)”.

Investigating the interaction between “age” and “sex” on the occurrence of AEFIs, we did not find any effect of such predictors (Supplementary Figure 3A and 3B). Supplementary Figure 3C shows the type and frequency of local and systemic AEFI according to sex and age strata. To further explore the possible impact of patients’ demographics on the occurrence of AEFIs, we performed a multivariable regression analysis using predictors “age” and “sex”; following both doses of vaccine, in RMD patients, the risk of AEFIs was significantly higher in younger patients and female patients (Fig. 2B). In HCs, an association between AEFI and younger age emerged only after the first dose (Fig. 2C).

The risk of AEFI was not influenced by ongoing treatment or previous SARS-CoV-2 infection (Supplementary Figure 4).

The efficacy of the SARS-CoV-2 vaccine was 99% at 5 months with only 2 RMD patients developing pauci-symptomatic COVID-19 after the first dose.

## Discussion

Showing a low frequency of disease flare and a similar risk of AEFI compared to healthy subjects, this study provides answers to the greatest concerns about the SARS-CoV-2 vaccine in patients with RMD.

As effective therapies for COVID-19 are still limited, vaccination is the best strategy for controlling SARS-CoV-2 infection. However, at the beginning of the third dose administration, many patients with RMD are still afraid of disease-related AEs and, most of all, vaccine-induced disease reactivation [5–8]. In general, non-live vaccines are recommended in patients with RMD, regardless of ongoing treatment with glucocorticoids and DMARDs [9, 10]. As for COVID-19, the American College of Rheumatology Guidance suggest that RMD patients should be offered COVID-19 vaccination (strong consensus), despite the theoretical risk of flare or disease worsening after SARS-CoV-2 vaccination (moderate consensus) [11, 12]. Data on the safety of SARS-CoV-2 vaccines in RMD patients are still scarce. The studies evaluating the immunogenicity of SARS-CoV-2 vaccines in RMD patients also briefly described the reactivation rate (ranging from 0 to 5%), and one multicentric study reported few cases of flare-up or new onset of immune-mediated diseases identified from the post-vaccination surveillance; indeed, Watad et al. described 17 flares (and 10 new onset) of immune-mediated diseases identified among the adverse event report forms from multiple academic centers located in 3 countries [13–16]. More recently, the Global Rheumatology Alliance (GRA) carried out a survey to investigate RMD patients’ perceptions and outcomes related to COVID-19 vaccines showing a 13.4% rate of flare after the vaccination in patients with different RMDs, with only about one third (4.6% of the whole cohort) requiring medication changes [8]. The GRA survey, however, was filled in by patients and no physician-led interview was performed. On the contrary, in this study, data on disease reactivation after SARS-CoV-2 vaccination was collected by rheumatologists showing a disease flare-up in 2.8% of immunized patients. The immunogenicity of mRNA vaccines relies on the ability of nucleic acids to act as a pathogen-associated molecular pattern that activates toll-like receptors, inducing a robust innate immune response leading to T and B cell activation [17]; thus, by eliciting a strong innate immune response, mRNA vaccines could theoretically activate also autoreactive cells, being a cause of concern for reactivation of autoimmune and inflammatory diseases. Currently, there are few data with SARS-CoV-2 vaccine and so far, there are no other licensed vaccines based on this technology. Patients treated with immunosuppressant were excluded from phase III BNT162b2 vaccine RCT; the trial included 118 patients with RMDs, of whom 62 patients in the vaccine group, without any data on the ongoing treatment [2]. Apparently, the outcome of the underlying disease was outside the scope of the efficacy trial. The trials evaluating immunogenicity and safety of mRNA vaccine for SARS-CoV-2, enrolling overall 1000 patients with different chronic inflammatory diseases, showed a low rate of disease reactivation during the observation periods ranging from 2 to 8 weeks of follow-up [13–15]. While Geisen et al. and Braun-Moscovici et al. reported no flare at all, Furer et al. reported a worsening of the symptoms of underlying RMD in 2.53% of patients after the first dose and 1.79% after the second one [13]. However, these 3 trials were designed to assess the immunogenicity of the vaccination, and the authors did not provide detailed data on the disease reactivation.

As in the present study, the GRA use a temporal criterion to define a disease flare-up—i.e., any worsening of the RMD lasting at least 2 days and occurring within 2 months from the first dose of vaccination [8]. In line with the previous reports, we showed an acceptable incidence rate—0.007 persons/month—of RMD reactivation in patients immunized with mRNA vaccine. After excluding other possible causes, and confirming the temporal relationship with the vaccination, only 3 out of the 126 evaluated patients showed a mild disease flare probably attributable to the vaccination. Indeed, none of the patients experiencing a flare changed the background therapy before the vaccination, and none of them reported any concurrent infection or any other suspected trigger responsible for the disease flare. In the GRA survey, the need of treatment adjustment was reported by 4.6% of the 2860 patients included in the study [8]; differently, only 2 out of 126 patients—0.02% of our cohort—needed symptomatic treatment, and their arthritis resolved after few days of glucocorticoids or non-steroidal anti-inflammatory drugs. Concerns about the safety of vaccines have been raised in sporadic reports and small open-label studies showing SLE reactivation after HPV vaccination, with a flare rate of 12.6% (95% CI, 0.04–0.21) [18]. Notably, we recorded only articular flares in patients with inflammatory chronic arthritis while no systemic nor major organ reactivation was reported in patients with connective tissue diseases and vasculitis. The survey published by GRA did not comment on the type of clinical manifestations reported by patients complaining of a flare nor on the type of underlying RMD [8].

Our data confirm the good tolerability of mRNA vaccines in patients with RMD who mainly complained of injection site reactions. As reported in RCTs, AEFI were more common in younger subjects; moreover, in RMD patients, AEFI were more common in females [2, 3]. We did not detect any difference in systemic AEFI between patients and controls while after the second dose, more patients than controls reported local reactions; overall, the probability of having local or systemic events was not influenced by the RMD nor by the ongoing treatment. Both local and systemic adverse events reported by RMD as well as HC were classifiable as minor reactions: mild-moderate events emerging within a few hours and resolving after a short period with no danger for the subjects. The two clinical trials investigating the immunogenicity of mRNA vaccination in RMD patients showed a lower percentage of both local and systemic adverse events compared to our cohort  [13, 14]. Most reactions were mild consisting in local pain  reported by 56–58% of patients and systemic reaction in a variable percentage of patients with fatigue being the most frequently reported (in up to 30%) [13, 14]. Furer et al. described two cases of death after the vaccination, out of the 686 patients who completed the study. The first one was the case of a woman with ANCA-associated vasculitis who died 3 weeks after the second dose of vaccine for a fulminant hemorrhagic skin vasculitis and sepsis; the second one was a man with PsA with a history of ischemic heart disease who died for a myocardial infarction 2 months after the second vaccine dose [14].

Finally, in line with data from RCTs, the efficacy of the SARS-CoV-2 vaccine was 99% at 2 months, with only 2 RMD patients developing pauci-symptomatic COVID-19 a few days after the first dose. In the studies by Furer and Braun-Moscovici, no cases of COVID-19 were reported among RMD patients who completed the vaccination [13, 14].

This study has some limitations. Patients were interviewed about AESI and were not asked to fill any diary. Similarly, reactivations of the disease were reported by patients and no physical examination or laboratory tests were performed to confirm the flare. However, the observational design and the physician-led interview enable to evaluate the safety and tolerability of mRNA SARS-CoV-2 vaccines in a real-life setting, providing relevant information for patients with RMD that were excluded from clinical trials. Moreover, we cannot draw a definitive conclusion on the efficacy of vaccination given the small sample size and the short-term observation.

## Conclusions

In conclusion, even if this study is limited by its observational nature, the results can reassure on the safety of mRNA SARS-CoV-2 vaccines, supporting one of the assumptions driving the American College of Rheumatology Guidance for COVID-19 vaccination in patients with RMS, “there is no reason to expect vaccine harms will trump expected COVID-19 vaccine benefits in RMD patients” [9]. Based on the observation of an overall good safety profile and, most of all, a low incidence rate of disease reactivation, patients with RMD who have not yet received SARS-CoV-2 vaccination should be encouraged to vaccinate.

## Supplementary Information

**Additional file 1: Figure S1**. Flow-chart of the phone call interview. **Figure S2**. Multivariable logistic regression analysis showing age- and sex-adjusted risk of systemic adverse events after I and II dose of vaccination in patients and controls. **Figure S3**. Occurrence of adverse events following immunization according to age and sex in patients and controls. **Figure S4**. Risk of adverse events following immunization in RMD patients according to ongoing treatment, previous SARS-CoV2 infection and treatment withdrawal prior to vaccination.

## Data Availability

Patients’ data are available from the corresponding upon reasonable request.

## Abbreviations

AE: Adverse event
AEFI: Adverse event following immunization
bDMARDs: Biological disease modifying anti-rheumatic drugs
COVID: Coronavirus disease
csDMARDs: Conventional synthetic disease modifying anti-rheumatic drugs
HC: Healthy controls
IQR: Interquartile range
JAK: Janus kinase
RMD: Rheumatic musculoskeletal diseases
RCTs: Randomized clinical trials
SARS-CoV-2: Severe acute respiratory syndrome coronavirus 2
