# Supplementary material for: Low frequency of disease flare in patients with rheumatic musculoskeletal diseases who received SARS-CoV-2 mRNA vaccine
Source: Arthritis Res Ther. 2022 Jan 11;24:21. doi: 10.1186/s13075-021-02674-w (PMC8748531; doi:10.1186/s13075-021-02674-w)
Supplement: Supplementary file 1 — Additional file 1: Figure S1. Flow-chart of the phone call interview. Figure S2. Multivariable logistic regression analysis showing age- and sex-adjusted risk of systemic adverse events after I and II dose of vaccination in patients and controls. Figure S3. Occurrence of adverse events following immunization according to age and sex in patients and controls. Figure S4. Risk of adverse events following immunization in RMD patients according to ongoing treatment, previous SARS-CoV2 infection and treatment withdrawal prior to vaccination. [file 13075_2021_2674_MOESM1_ESM.docx]

**Supplementary Figure 1. Flow-chart of the phone call interview.**

The list of symptoms recorded after both doses includes local reactogenicity, arthromyalgia, malaise, fever, lymphadenitis, other.

**Supplementary Figure 2. Multivariable logistic regression analysis showing age- and sex-adjusted risk of systemic adverse events after I and II dose of vaccination in patients and controls.**


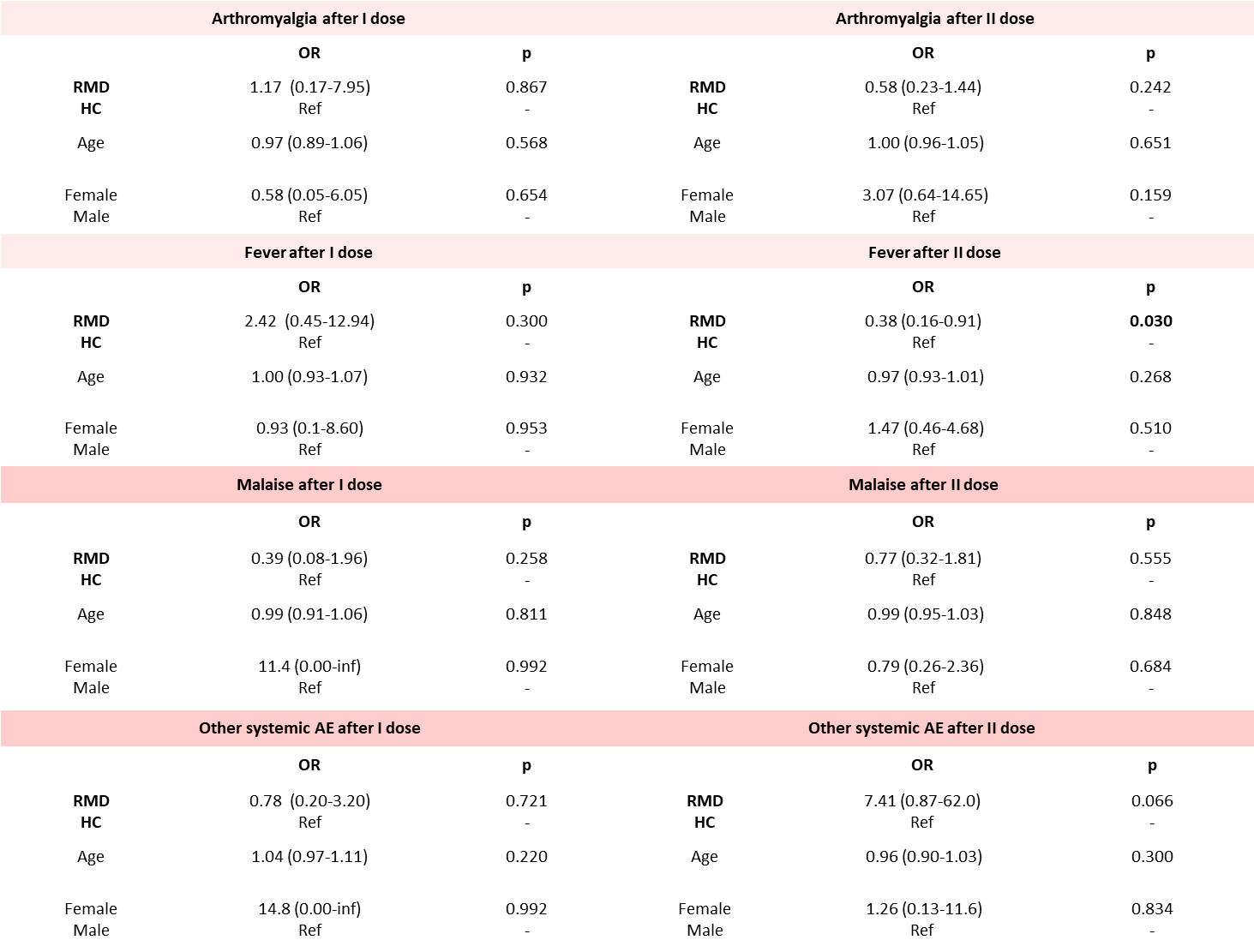


RMD = rheumatic musculoskeletal disease, HC = healthy controls.

**Supplementary Figure 3. Occurrence of** **adverse events following immunization according to age and sex in patients and controls.**

**
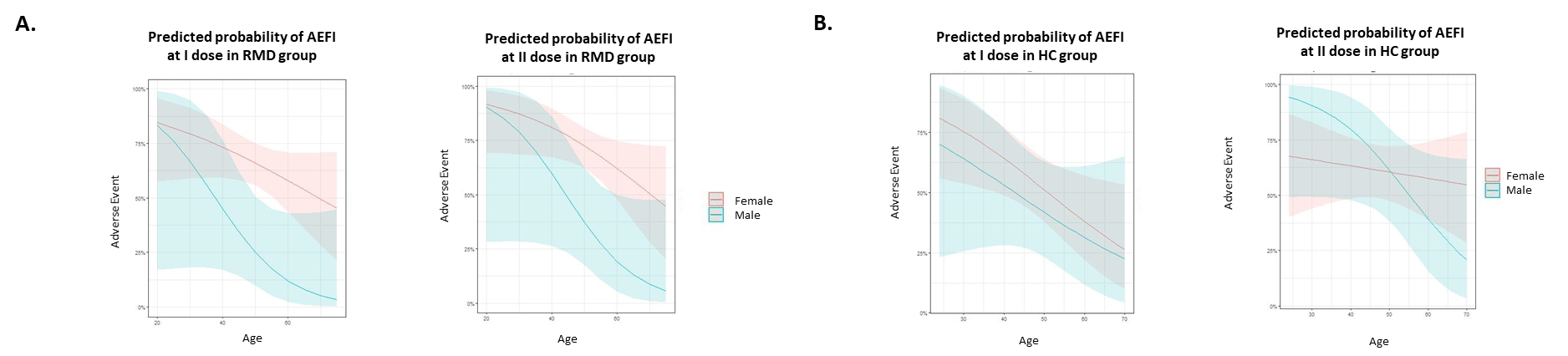
**


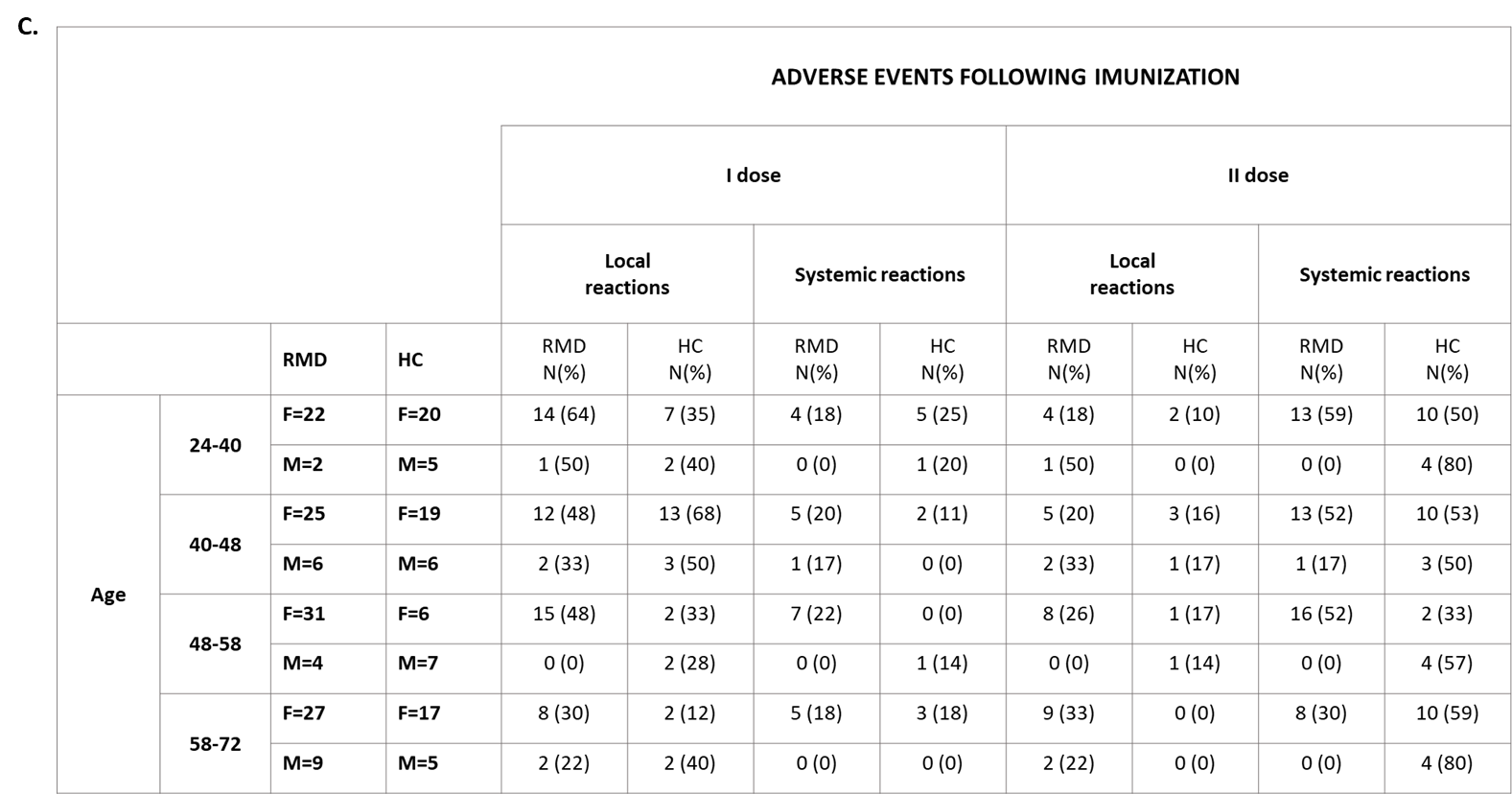


Panel A and panel B represent the interaction model including age and sex in RMD patients (A) and healthy controls (B). Panel C summarizes the proportion of male and female patients and controls complaining of local and systemic reaction, according to age strata.

AEFI = adverse events following immunization, RMD = rhematic musculoskeletal diseases, HC = healthy controls

**Figure 4. Risk of adverse events following immunization in RMD patients according to ongoing treatment, previous SARS-CoV2 infection and treatment withdrawal prior to vaccination.**

**
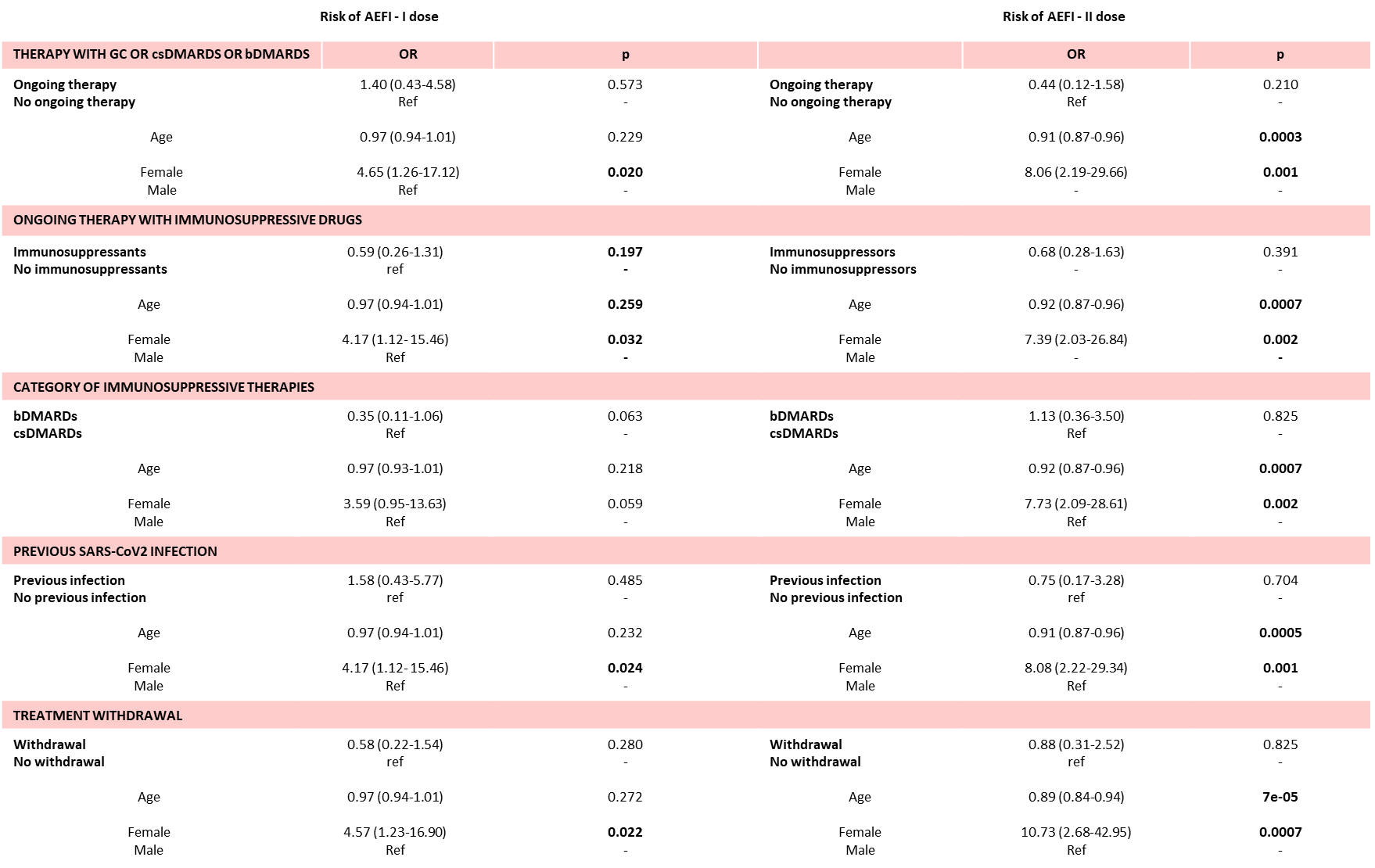
**

RMD = rheumatic musculoskeletal diseases, GC = glucocorticoids, csDMARDs = conventional synthetic anti-rheumatic drugs, bDMARDs = biological anti-rheumatic drugs
